# Medical Teaching in Paris, &c.

**Published:** 1853-06

**Authors:** D. J. Cain


*Editorial Correspondence of the Charleston Medical Journal and Review.*

PARIS, MARCH 5th, 1853.

*Rue de Seine, St. Germain.*

Medical Teaching in Paris; Epidemic of Typhoid Fever, Treatment of; M. M. Briquet, Bouilland and Louis; Post Mortem; Statistics; Suicides; Asiatic Cholera; Puerperal Fever; M. Trosseau; Treatment of Rheumatism by Quin ine and Veratrine; Service of M. Guersant at Hospital for sick children; Surgical Operations; Cervical Rheumatism; Erectile Tumour; *Cours d'Accouchement* in Paris; Sages-femmes; Recent expression of the views of M. Coste on Embryology; Honors conferred upon Americans, &c.

D. J. CAIN, M.D.:

*Dear Sir:*—As I have quite a *melange* to introduce, it is best, perhaps, to address this communication to you personally. Good reasons exist for the non-appearance of one in the March number, but these shall be reserved for your private ear.

We are of the midst of a period in the year when medical science is pursued with more vigor than at any other—when the Professors at the *Ecole de Medecine* are in the midst of the winter campaign, some of them with an audience of a hundred, others with upwards of a thousand. Now also there are a legion of Lecturers, public and private, who occupy almost every hour of the twenty-four at the *Ecole Practique*, and in private rooms in the neighborhood. Besides, each Interne or Chief of the Cliniques of any note, has his private class of four or five at the Hospitals, who, for twenty-five to fifty francs per month, enjoy the privilege of making visits and examinations, and of studying, auscultation and percussion with him during the evening. The College of France, the Sorbonne and the Jardin des Plantes, have each also its corps of Lecturers and Professors, who discourse on general subjects:—Law, Theology, Ancient and Modern Literature, &c.; but among whom, nevertheless, are found M. M. Geoffroy, Saint-

Hiliare, Flourens, Milne Edwards, Coste, and, during the summer, M. M. Orfila, Magendie and Bernard. The latter has just completed an extremely interesting private course on Experimental Physiology with vivisections, and we had intended replacing this letter with a portion of our notes taken during their delivery. A friend, however, having furnished us with some of his own, we willingly send them, possibly continuing the subject ourselves at a future time when more leisure shall be allowed us. We may add that M. Ludovic's Demonstrations at the school for Practical Anatomy have also been quite instructive. He was, you are aware, the collaborator of Bongery in the preparation of his Plates, and one of the most adroit dissectors in Paris.

Among those first alluded to, M. M. Denouvilliers and Orfila attract much the largest number of followers. We have for two months been attending M. Malgaigne, at the School of Medicine, during which time he has confined himself to the surgical treatment of maladies of the Eye; M. Denouvilliers has consumed the same period in the introductory portions of general Anatomy, having only within a few days commenced with a particular region. One may judge by this how minutely they pass over the subjects allotted them, in my opinion consuming a disproportionate amount of the space in treating of only a fractional part of what demands consideration, and what the student will require. However, they allow him more years in which to earn his diploma here than in the United States, and can thus afford to be *thorough*. We are aware that it is impossible for the same condition of things, however desirable, to exist in our country, until Governments, whether State or General, shall forcibly assume the direction and expenses of certain species of educational establishments, and wrest from the hands of one set that may be unfit, the power to confer diplomas upon others equally incapable of assuming the responsible duties of the physician who *suddenly* goes out to cure a thousand various maladies, each with its own peculiar history, its characteristic phases and relations. Thus aiding the well selected power which instructs and which confers; liberating the professors from the thraldom of pecuniary competition, and leaving them to that of intellectual; thus enabling them to direct all their energies in elevating and ennobling the profession. If the reward of the professor depends upon, and is in proportion to, the number of those who enrol in his school, he must at times enter into strife with ignoble rivals, the worst of human passions mingle in the contest, underbidding and depreciation ensue, and the profession sways backward as often as it does forward, instead of moving directly and constantly upward in the path of progress.

An epidemic of Typhoid Fever is very prevalent in Paris at present, and it is seen in all the Hospitals. In these it is quite interesting to witness the variety of treatment adopted by each physician of note, as his medical tendencies incline; that pursued respectively by M. M. Boullaud, Briquet and Louis, whom I have followed, presents many points of difference, and it has been difficult to determine on which side the scale of success leans. We hope, however, to be able to send a paper by a medical friend who has watched closely the service of the former, and who can furnish some testimony with respect to his mode of employing the lancet in this disease. M. Louis very generally administers seltzer water, vinous lemonade, and adopts, to a certain extent, the expectant system. M. Briquet at La Charite pursues what appears to me rather a middle course. He abstracts blood in the early stage of some cases which seem peculiarly fitted for it. I may observe here that I have also heard M Trousseau say, that even as late as during the second and third septenary period, he was in the habit of bleeding to prevent the increase, or destroy the congestion of the lungs which so often exhibits itself, its approach and presence being of course easily discoverable by the physical signs. M. Briquet relies with much confidence, (and my own observation in his wards can sustain him,) upon the application of cups and scarification to the mastoid region during delirium and coma of Typhoid. It completely checks the increase of these dangerous symptoms, as I have repeatedly witnessed. He does not give mercury internally, but applies plasters of mercurial ointment over the entire abdomen until the secretions become active, or the tongue and skin exhibits the desired moisture. This method of using the remedy might more easily reconcile itself to those who so much doubt respecting the advantages of its internal administration. He gives ordinary Bordeaux wine, during the period of convalescence. These means, thus generally expressed, are often associated in his practice with the use of ice, administered in every possible way, and through every channel—bladders filled with it are applied to the scalp, it is dissolved in the mouth and given with enemata. One of his Internes mentioned to me this morning, that he had lost but one case within three weeks. There are between twenty and fifty sick of the disease in each service of the Hospitals, and few of these fail to present gargouillement, the characteristic rose mark and sudamina.

As the subject of typhoid fever has been much discussed of late at the South, and in the pages of this Journal, it may not be thought useless to consider the following autopsy which I witnessed at La Charite a few days since. It is instructive as indicating the characteristic march of the disease in an individual in whose system it was not modified by medicines. The subject, a man of about 33 years of age, of good conformation when he entered the Hospital, presented every favorable condition save the existence of the endemic; this had seized upon him as it had upon most of those who had within the last year or two moved from the country to Paris, and who did not live under favorable hygienic conditions. He had been bled, but absolutely no medicines were taken with the exception of *tisans* and mucilaginous drinks. Notwithstanding, the entire lining membrane of his *stomach* was intensely red and exhibited all the traces of severe inflammation: there was some exudation of plastic lymph, and the particles of coloring matter seemed infiltrated beneath the mucous coat; when scraped off with a sharp bistoury, the surface beneath was white. Thirty of Payer's glands were enlarged and inflamed, decreasing in size from the commencement of the colon, until they disappeared in the small intestines. The largest were about an inch and a quarter in diameter, with elevated edges, presenting, as M. Briquet remarked, precisely the appearance of carcinoma; the surface of those situated most inferiorly exhibited the yellow summit, indicating the commencement of gangrene. No other portion of the intestinal canal was diseased, if we exclude the above enlarged mesenteric glands. The *spleen* was four times the ordinary size, though it did not appear that the deceased had suffered from intermittent fever. The cavities of the *heart* contained some fibrinous deposits, and its muscular tissues were soft. The lungs showed previous inflammation of the bronchia, and the lower lobes were still engorged; these did not compromise the life of the individual, as quite a large portion was spongy and elastic. The consistence of the *brain* was firm, no fluid was found in the ventricles, but there was venous engorgement on its surface. The *liver* and other organs remained quite natural.

Four cases of Asiatic Cholera appeared at Hotel Dieu during the month of January—so said the journals and the physicians! I heard M. Louis observe, a few mornings since, that the diarrhœa or cholerine, which had since existed to a certain extent, had not respected any class of the patients, not excepting those with typhoid fever. Before leaving the subject of this latter affection, which is now on the decline, I append a few statistical items which I have selected and re-arranged from a mass of valuable matter contained in the *Moniteur*. It may afford data for comparative estimates in the United States.

On the first of March there were 1.422 sick of the disease, 4,344 having been the maximum of those sick in the Hospitals. At the same time the number of the sick, generally, has descended from 6,735 to 6,618. From the 19th to 27th of February, (9 days,) 131 had died of 1,253 sick of typhoid in hospitals, or about 1 in in 100—a proportion less than has characterized any previous epidemic. The winter has been a very wet one, the Seine having been higher than for many years past, and snow having fallen frequently within the last month.

During the year 1852, there were 29,873 deaths in Paris. 29,664 had the relations of sex, age and disease indicated. Of these 16,-220 were males, and 14,444 females. In 1851, there were 29,709 deaths; 15,110 males, 14,599 females. The most fatal period of life was under three months; the least, between six and eight years. Among the 29,664 deaths, maladies of the chest, digestive apparatus, and Typhoid Fever occurred most frequently. Of Pulmonary Phthisis, there died 2,073 males, 2,038 females; of Pneumonia, 1,279 males, 1,346 females; of Pulmonary Catarrh, 849 males, 955 females; of Enteritis, 1,395 males, 1,365 females; of Typhoid Fever, 603 males, 503 females. The temperature was quite elevated during the whole year, though there were few atmospheric variations. Of the 29,873, 10,157 were in Hospitals, remainder *a domicile*, a word which may well be retained to mark those sick in the city, but not in the medical establishments. In 1829, when statistical researches commenced to attract greater attention, there were 17,101 deaths *a domicile*, and 9,884 in the Hospitals, the population then being 776,241.—In 1832, the numbers rose to 30.843 dead *a domicile*, 15,481 in Hospitals. This increase is accounted for by the presence of Asiatic Cholera. 1814, the year of the war, was the most fatal, there being 24,197 deaths, the severest epidemic of Typhoid ever experienced in Paris, contributed to swell the number of victims.—The most melancholy reminiscences are said even now to attach to this sad epoch, which elicit a great deal of generous sympathy.

We observe, from comparative observations, that the number of persons carried to the Morgue annually varies from 250 to 320 males, from 40 to 60 females and from 15 to 25 new born—the males generally being to the females in the ratio of about five to one. From the year 1819 to 1828, there were transported to this spot 2,765 cadavers, of which 485 were female. To show the depressing effect of an epidemic, I will cite for comparison, the two years 1831 and 1832, when the population was of course nearly the same. In the former there were 232 bodies of males, and 67 females and 5 new born. In 1832, the year of cholera, 1,007 of males, and 225 females. It must be remembered that these include not only suicides but those also destroyed by accidents. It is remarkable that among the suicides of the past year there were 4 boys between 8 and 15 years of age, and 6 girls; 13 boys of from 15 to 20 years, and 9 girls. There were, also, 8 men and 4 women of from 70 to 80 years of age.

On account of the severity of Peuperal Fever at the Clinique of the School of Medicine, M. Paul Dubois has had to discontinue the reception of parturient women. The epidemic was attended with considerable mortality. They have not, however, been able to practice fumigations or purification, as the *Bureau of Admin-*

*istration* have been forced to occupy the beds with Typhoid patients—so great is the need at present of accommodation for the sick.

Our readers are aware that M. Trousseau has been appointed in the place of M. Chomel, at Hotel Dieu, where his cliniques are very popular indeed. It is a source of extreme regret to many that he has thus been forced to leave the *Hopital des Enfants' Maladies*, and to discontinue his usual course of Lectures during the summer on Meteria Medica and Therapeutics. The treatment of acute Rheumatism by Quinine, commenced by Briquet, and to a certain extent discontinued, on account of the doubtful character of the termination of one or two cases in which large quantities were administered, has been revived of late, and the impression respecting it gains favor daily. I have seen M. B. give it with good effect quite often. M. Valleix employs and recommends it at La Pitie, and I hope to be able to send a paper on the subject by a gentleman who attends his *Services* and Lectures. One gramme (15 grains) is administered once or twice a day, and the patient kept partly quininized. The gravity of the usual symptoms daily decrease, and it seems to be not at all hostile to cordiac complications. M. Trousseau, in commenting upon this, and expressing his favorable impression, alluded also to the expense attending the use of this agent, accompanying it at the same time with the relation of a remarkable instance of recovery from acute articular Rheumatism, which was then in his wards. It was true, he added, that it was as yet an isolated case, but the repetition of the means used might give equally favorable results. It followed the employmennt of Veratrine after the method recommended by M. Daniel( ?). The patient, a woman under middle age, suffered intense pain in the articulations, high fever with endo-carditis exhibiting itself by a derangement of the first sound at the base of the heart—(sigmoid valves of aorta)—with a souffle prolonged into the large vessels, and accompanied with an alteration in the left ventricle also. In 62 hours she was absolutely cured, leaving not the slightest trace of fibrile excitement, the presence of a slight souffle alone remains. One half of a millegramme (about 1-10th of a grain ?) was given in the form of pill, each day increasing the quantity, and, when the pains were relieved, continuing it in the same dose every second day. Its influence on the circulation in this case was prodigious, to use M. Trousseau's expression; the pulse fell to 42, and when I heard of the patient two days after, it was still at this reduced rate. Veratrine, we are aware, is one of the active principles of colchium, and it is, therefore, not surprising that it should possess some power in acute Rheumatism, more particularly in those dependent upon the arthritic diathesis, gout, etc.

(*Concluded next Month.*)

